# M-currents (Kv7.2-7.3/KCNQ2-KCNQ3) Are Responsible for Dysfunctional Autonomic Control in Hypertensive Rats

**DOI:** 10.3389/fphys.2016.00584

**Published:** 2016-11-29

**Authors:** Torill Berg

**Affiliations:** Division of Physiology, Department of Molecular Medicine, Institute for Basic Medical Sciences, University of OsloOslo, Norway

**Keywords:** hypertension, M-channels, parasympathetic ganglia, heart rate, 3, 4-diaminopyridine, Kv7.x, catecholamines, vascular tension

## Abstract

Autonomic dysfunctions play important roles in hypertension, heart failure and arrhythmia, often with a detrimental and fatal effect. The present study analyzed if these dysfunctions involved M-channels (members of the Kv7/KNCQ family) in spontaneously hypertensive rats (SHR). Cardiac output and heart rate (HR) were recorded by a flow probe on the ascending aorta in anesthetized SHR and normotensive rats (WKY), and blood pressure (BP) by a femoral artery catheter. Total peripheral vascular resistance (TPR) was calculated. XE-991 (Kv7.1-7.4-inhibitor) reduced resting HR in WKY but only after reserpine in SHR. XE-991 increased TPR and BP baseline in both strains. Retigabine (Kv7.2-7.5-opener) reduced HR, TPR and BP, also after reserpine. Depolarization induced by 3,4-diaminopyridine (3,4-DAP), a voltage-sensitive K^+^ channel (Kv) inhibitor, activated release of both acetylcholine and norepinephrine, thus activating an initial, cholinergic bradycardia in SHR, followed by sustained, norepinephrine-dependant tachycardia in both strains. XE-991 augmented the initial 3,4-DAP-induced bradycardia and eliminated the late tachycardia in SHR, but not in WKY. The increased bradycardia was eliminated by hexamethonium and methoctramine (M2muscarinic receptor antagonist) but not reserpine. Retigabine eliminated the increased bradycardia observed in reserpinized SHR. XE-991 also increased 3,4-DAP-stimulated catecholamine release, but not after hexamethonium or reserpine. Conclusions: M-currents hampered parasympathetic ganglion excitation and, through that, vagal control of HR, in SHR but not WKY. M-currents also opposed catecholamine release in SHR but not in WKY. M-currents represented a vasodilatory component in resting TPR-control, with no strain-related difference detected. Excessive M-currents may represent the underlying cause of autonomic dysfunctions in hypertension.

## Introduction

In hypertension and heart failure, heart rate (HR) is typically controlled by a sympathetic hyperactivity and parasympathetic hypoactivity (Binkley et al., 1991; Thrasher, 2005), and a high resting HR is the most reliable predictor of cardiovascular morbidity in man (Palatini, 1999). With a failing vagal control of HR, sympathetic hyperactivity may lead to hypertensive left ventricular hypertrophy (Schlaich et al., 2003), an independent predictor of morbidity and mortality (Levy et al., 1990). Moreover, abnormalities in the vagal control of HR may be directly responsible for a poor outcome in myocardial infarction (Kleiger et al., 1987), and autonomic dysfunctions also contribute to the pathology of atrial fibrillation (Park et al., 2012). Dysfunctions in the balance between parasympathetic and sympathetic control of HR is therefore detrimental, and may result in a fatal outcome.

In a recent study, hampered vagal control of HR in spontaneously hypertensive rats (SHR) was located to a reduced parasympathetic ganglion transmission (Berg, 2015). Evidence in animal and man indicated that the parasympathetic ganglia acted as a bottleneck to efferent vagal traffic also in heart failure (Bibevski and Dunlap, 2011). Neurons are activated by depolarizing action potentials, which open voltage-sensitive Ca^2+^ channels (Ca_V_). The subsequent increase in intracellular Ca^2+^ precipitates release of transmitter from vesicles in the presynaptic nerve terminal. K^+^ channels have a hyperpolarizing effect and therefore oppose depolarization and excitation. M-type, voltage-sensitive K^+^ channels of the Kv7 family, mainly Kv7.2-7.3 (KNCQ2-KNCQ3) (Wang et al., 1998), are known to suppress neuronal excitability and transmitter release throughout the nervous system (Brown and Passmore, 2009). It was therefore hypothesized that excessive M-currents hampered parasympathetic ganglion transmission and in that manner interfered with vagal control of HR in SHR.

**Figure 1:**
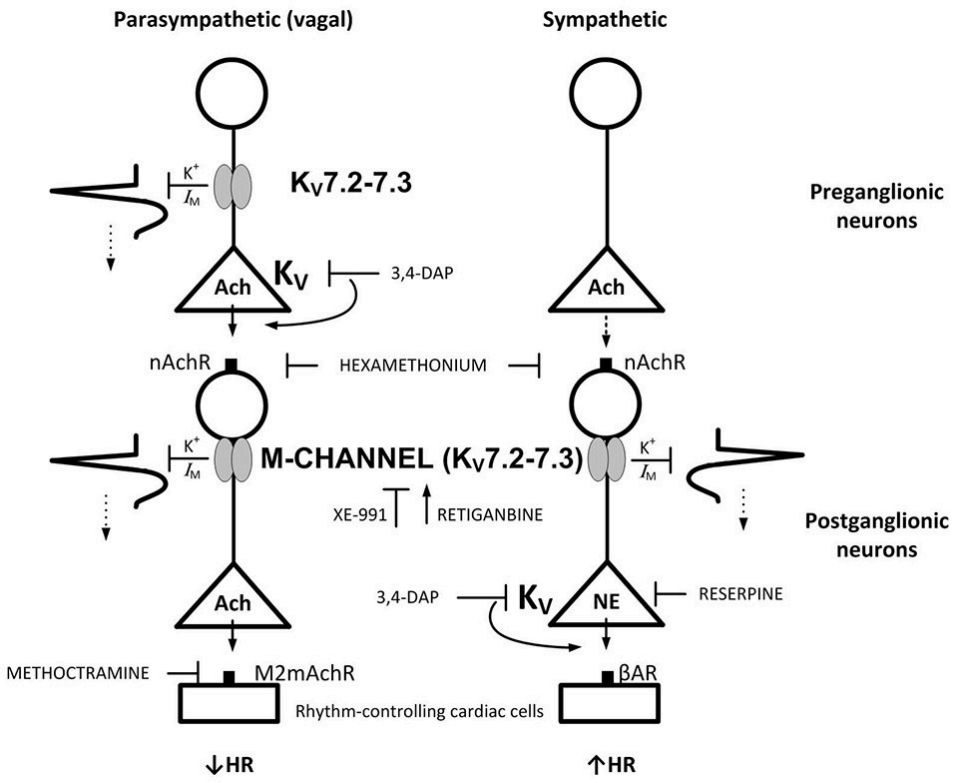
**M-currents are responsible for the vagal dysfunction and failing autonomic control of HR in SHR**. By inhibiting Kv, 3,4-DAP will induce Ach release primarily from preganglionic parasympathetic neurons and norepinephrine from sympathetic nerve endings as indicated, and through that induce bradycardia followed by tachycardia.(Berg, 2015) M-currents (Kv7.2-7.3) hamper neuron excitability. In the parasympathetic branch, the M-channels may be located both in the preganglionic and postganglionic neurons, whereas a postganglionic localization seemed likely in the sympathetic branch. Drug names indicate site of main action. Arrows and blunted arrows indicate stimulatory and inhibitory actions, respectively.

Analysis of the interaction between sympathetic and parasympathetic control of HR requires both systems to be activated simultaneously. This is present in conscious individuals, where Frequency Domain analysis of for instance respiratory arrhythmia can be used to indicate changes in parasympathetic activity. However, this arrhythmia depends on vagal reflexes, and such reflexes are disturbed by the anesthesia (Berg and Jensen, 2011). However, dual activation of both parasympathetic and sympathetic autonomic branch can be achieved in the anesthetized rat by 3,4-diaminopyridine (3,4-DAP) (Berg, 2015). By blocking presynaptic, voltage-sensitive K^+^ channels (Kv) (Vlcková and Stolc, 1990), 3,4-DAP induces neuronal depolarization, opening of voltage-sensitive Ca^2+^ channels (Ca_V_) and, thus, transmitter release. 3,4-DAP therefore mimics the events occurring during nerve stimulation, and may activate transmitter release in sympathetic and parasympathetic ganglia, as well as from peripheral autonomic nerves (Figure 1). An IV injection of 3,4-DAP therefore induced an initial parasympathetic, atropine-sensitive bradycardia, followed by a sustained tachycardia elicited by norepinephrine released from peripheral sympathetic nerve terminals (Berg, 2015). The parasympathetic component was greater in SHR than in their normotensive controls (WKY) (Berg, 2015). This difference was located to the parasympathetic ganglia (Figure 1) (Berg, 2015). If the hyperpolarizing M-channels are closed, neurons are expected to be more excitable by the depolarization occurring during an action potential. By studying effects on HR and catecholamine release, the present study therefore tested if the role of M-channel in the control of autonomic nerve excitability in SHR differed from that in WKY (Figure 1), at rest and during 3,4-DAP-induced depolarization.

Members of the Kv7-family, i.e., Kv7.1, Kv7.4, and Kv7.5 (Mackie et al., 2008), as well as 3,4-DAP-sensitive Kv-channels (Berg, 2002, 2003), have been detected in rat artery vascular smooth muscle cells (VSMC), where they oppose depolarization and the entry of Ca^2+^ through Ca_V_ and in that manner oppose vasoconstriction. Reduced activation of these currents in VSMC may therefore contribute to the elevated total peripheral vascular resistance (TPR) characterizing hypertension. The second goal of the present experiments was therefore to test if Kv7-currents influenced TPR and blood pressure (BP) differently in SHR and WKY.

## Methods

### Surgical procedure

All experiments were approved by The Norwegian Animal Research Authority (NARA), project license no FOTS2914 and FOTS8464, and conducted in accordance with the European Directive 2010/63/EU. Breeding of WKY and SHR, originally obtained from formally legacy Harlan, now Envigo RMS, Bicester, Oxfordshire, UK, was done in-house. The rats were kept on a 12/12 h day/night cycle and fed with Teklad Global 18% Protein Rodent Diet (Teklad Diets, Madison, WI, USA) containing 0.2% Na^+^. The SHR (Okamoto, SHR/NHsd strain, *n* = 88) and WKY (Wistar Kyoto, *n* = 36) included in this study, weighed 270 ± 3 g and 267 ± 5 g, respectively, and were 12.2 ± 0.1 and 12.7 ± 0.2 weeks old. The rats were anesthetized with pentobarbital (65–75 mg/kg IP), and the level of anesthesia was tested by non-responsiveness to pinching between the toes. When satisfactory anesthesia was established, it remained throughout the experimental period without further supply.

After tracheotomy, a heparinized catheter was inserted into the femoral artery to measure systolic (SBP) and diastolic (DBP) BP and HR, which at this time was not influenced by the subsequent, artificial ventilation. The arterial catheter was connected to a SensoNor 840 transducer (SensoNor, Horten, Norway). After the starting BP and HR had been recorded, the rats were connected to a positive-pressure ventilator and ventilated with air throughout the experiment. The thoracic cavity was entered through the third intercostal space, and a 2SB perivascular flow probe, connected to a T206 Ultrasonic Transit-Time Flowmeter (Transonic Systems Inc., Ithaca, NY, USA), was placed on the ascending aorta for continuous measurement of cardiac output (CO, i.e., without cardiac flow) and from now on also HR. The thoracic cavity was subsequently closed with a suture. The pressure transducer and flowmeter were coupled to an amplifier and computer to continuously store and analyze the data. Mean arterial BP (MBP = SBP-DBP/3+DBP) and TPR (MBP/CO) were calculated. Body temperature was maintained at 37–38°C by external heating, monitored by a thermo sensor inserted inguinally into the abdominal cavity. The arterial catheter was subsequently flushed with 0.15 ml buffered saline (PBS; 0.01 M Na-phosphate, pH 7.4, 0.14 M NaCl) containing 500 I.U./ml heparin. The animals were injected as needed with 1–2 ml PBS IV to stabilize BP. A period of 10 min was then allowed before the first experimental drug was injected. Drugs were dissolved in PBS and administered as bolus injections through a catheter in the femoral vein (0.6–1.3 ml/kg), unless otherwise indicated.

Blood was collected from the arterial catheter at the end of the 25-min 3,4-DAP-observation-period to determine the plasma catecholamine concentrations. 3,4-DAP-induced autonomic activation also stimulates salivary secretion. Whole saliva was collected with a pipette from the oral cavity throughout the 3,4-DAP-observation period, and saliva volume was estimated by weight.

### Experimental protocols

WKY and SHR control rats were pre-treated with an IV sham injection of vehicle (PBS) and injected 10 min later with the Kv-blocker 3,4-DAP (34.5 μmol/kg, IV), which does not cross the blood-brain barrier, and which will activate the autonomic nervous system (Berg, 2015). 3,4-DAP does not interfere with Kv7 (Yeung et al., 2007). In the WKY and SHR experimental groups, the PBS-sham injection was substituted with either Kv7- blockers, i.e., XE-991 (Kv7.1-7.4, 2.2 μmol/kg, −10 min) (Yeung et al., 2007) and chromanol 293B (Kv7.1 (K_s_), 3 μmol/kg, −10 min, not followed by 3,4-DAP in WKY) (Yang et al., 2004) or Kv7-openers, i.e., regitabine (Kv7.2-7.5, 4.3 μmol/kg, −30 min) (Wickenden et al., 2000) and ICA-27243 (Kv7.2-7.3, 11.2 μmol/kg, −10 min, tested in SHR only) (Qi et al., 2011).

XE-991 was found to influence the HR-response to 3,4-DAP in SHR but not in WKY. The ganglion and parasympathetic and sympathetic involvement in these changes were therefore further analyzed in SHR only. The nicotinic receptor (nAchR) involvement was studied by pre-treatment with the ganglion blocker/nAchR antagonist hexamethonium (37 μmol/kg, −10 min, IV) (Berg, 2005), administered either alone or prior to XE-991. Influence of norepinephrine release was analyzed by pre-treatment with reserpine (8.2 μmol/kg, −48 and −24 h, IP) (Berg, 2002), which depletes sympathetic nerve terminals of norepinephrine. Postsynaptic muscarinic AchR (mAchR) in the heart are of the M2-subtype, and are inhibited by the M2mAchR antagonist methoctramine (Wess et al., 1988). Parasympathetic involvement was therefore demonstrated by methoctramine (0.41 μmol/kg, −10 min, IV).

The effect of hexamethonium and reserpine alone has been tested previously in WKY (Berg, 2015) and was not repeated here. However, only the non-selective mAchR antagonist atropine has been tested before in WKY, and WKY pre-treated with methoctramine were therefore included in the present protocol.

### Measurement of plasma catecholamines

1.5 ml blood was collected from the femoral artery into tubes containing 45 μl 0.2 M glutathione and 0.2 M ethylene glycol-bis(2-aminoethylether)-N,N,N′,N′-tetraacetic acid (EGTA) (4°C). Plasma was stored at −80°C until catecholamine concentrations were determined using 400 μl plasma and the 5000 Reagent kit for HPLC analysis of Catecholamines in plasma from Chromsystems GmbH, Munich, Germany, as described by the manufacturer. The samples were run on a Shimadzu monoamines analyzer system, using an isocratic flow rate of 0.8 ml/min, and an electrochemical detector (Decade II) and a SenCell electrochemical flow cell (Antec Leyden, Zoeterwoude, The Netherlands).

### Drugs

Pentobarbital was obtained from The Norwegian National Hospital, Oslo, Norway; retigabine (ethyl-(2-amino-4-(4-fluorobenzylamino)-phenyl)carbamate dihydrochloride) and XE-991 dihydrochloride (10,10-bis(4-pyridinylmethyl)-9(10H)-anthracenone) from MedChem Express, Princeton, NJ, USA; chromanol 293B (trans-N-[6-Cyano-3,4-dihydro-3-hydroxy- -2,2-dimethyl-2H-1-benzopyran-4-yl]-N-methyl-ethanesulfonamide) from Tocris Bioscience, Bristol, UK; ICA-27243 (N-(6-chloro-pyridin-3-yl)-3,4-difluoro-benzamide) from Alomone Labs, Jerusalem, Israel; and methoctramine hydrate (N,N'-bis[6-[(2-methoxybenzyl)amino]hexyl]-1, 8-octanediamine tetrahydrochloride) from Sigma Chemical Co., St. Louis, MO, USA.

### Statistical analyses

Each group comprised 6-9 rats. All results are presented as mean values ± s.e.mean. The cardiovascular data recorded throughout the experiments were averaged every minute, except during the initial response to PBS, XE-991, chromanol, retigabine, ICA-27243 or 3,4-DAP, where data were averaged every 7th heart-beat. The latter HR-response was recorded at the same time as the highest/lowest TPR-response was detected, even if a different HR was seen before that. The cardiovascular response to pre-treatment, baselines prior to 3,4-DAP, saliva volume and the plasma catecholamine concentrations were evaluated over-all by one-way ANOVA, including all groups within each strain. When the presence of group differences was indicated, these were located by two-tailed, two-sample Student's *t*-tests for parametric data, and by Kruskal-Wallis tests for non-parametric data. The 3,4-DAP-response curves included values recorded at the initial TPR peak-response, i.e., at about 1–1.5 min, and during the late response, i.e., at 10, 15, 20, and 25 min. Since TPR is determined by the vessel radius in the fourth power, changes in TPR were expressed in percent of before values. The 3,4-DAP-response curves were analyzed using Repeated Measures Analyses of Variance and Covariance, first as over-all tests including all groups within each strain, and subsequently between groups or for each group separately. Group differences (two-tailed, two-sample Student's *t*-tests or Kruskall-Wallis tests) were subsequently located at specific times, i.e., during the initial response and at the end of the experiment. Testing proceeded only when the presence of significant responses, differences and/or interactions was indicated. The *P*-value was for all tests and each step adjusted according to Bonferroni, except for saliva volume and the plasma catecholamine concentrations, where *P* ≤ 0.05 was considered significant.

## Results

### The cardiovascular response to Kv7-inhibitors/openers at rest

At the beginning of surgery, i.e., before the rats were connected to the ventilator, MBP was 79 ± 3 and 133 ± 6 mmHg in WKY and SHR, respectively (*P* < 0.001, pooled data for all non-reserpinized rats). HR at this time was 306 ± 7 and 389 ± 5 bpm, respectively (*P* < 0.001). Prior administration of reserpine in SHR (not tested in WKY) lowered these parameters to 95 ± 3 mmHg and 311 ± 5 bpm (*P* < 0.001). After being connected to the positive-pressure ventilator and thoracotomized, MBP was reduced (*P* < 0.001) to 61 ± 2 mmHg in WKY and to 79 ± 3 mmHg in SHR, since SHR were more sensitive than WKY to the reduced venous return to the right heart due to the ventilator-dependent intrathoracic pressure. HR at this time was 339 ± 8 bpm in WKY (*P* = 0.003 compared to before) and 398 ± 5 bpm in SHR (*P* = NS). The PBS-sham-injection had little effect on the cardiovascular baselines, and MBP, HR, and TPR prior to 3,4-DAP were higher in SHR than in WKY (Table 1).

**Table 1:** **Cardiovascular baselines after pre-treatment, and the response to pre-treatment below in parenthesis**.

**Pre-treatment**	**WKY**				**SHR**			
	**MBP mmHg**	**HR bpm**	**CO ml/min**	**TPR mmHg/ml/min**	**MBP mmHg**	**HR bpm**	**CO ml/min**	**TPR mmHg/ml/min**
PBS	61±4	339±12	28±1	2.2±0.1	89±10^*^	388±12^**^	31±3	2.9±0.2^**^
	(−3±2)	(−16±3)	(0±1)	(−0.2±0.0)	(−4±4)	(−11±8)	(2±1)	(−0.3±0.1)
PBS after reserpine^a^					64±4^†^	361±12	27±2	2.4±0.1
					(−4±2)	(−16±5)	(−1±0)	(−0.1±0.0)
Methoctramine+PBS	60±2	366±7	31±2	2.0±0.1	83±6	458±11^†††^	21±2^†^	4.0±0.3^††^
	(−1±2)	(32±12)^††^	(3±1)	(−0.3±0.1)	(12±7)	(50±17)^††^	(−1±1)	(0.6±0.2)^††^
Hexamethonium					53±2^††^	345±19^†^	25±1	2.9±0.2
					(−33±7)^††^	(−67±8)^†††^	(−1±1)	(−1.4±0.2)^††^
XE-991	48±3^†^	290±22	32±6	1.8±0.3	65±3^†^	384±13	24±1	2.8±0.2
	(−7±5)	(−43±24)	(5±3)	(−0.4±0.2)	(−14±5)	(−21±15)	(2±1)	(−0.8±0.2)
XE-991 after reserpine^a^					61±4^†^	340±10^†^	31±1	2.0±0.1^††^
					(−12±3)	(−41±12)	(4±1)	(−0.7±0.1)
Methoctramine+XE-991					66±8	442±8^††^	21±2^†^	3.2±0.2
					(−16±7)	(50±6)^†††^	(0±1)	(−0.8±0.5)
Hexamethonium+XE-991					49±3^††^	334±10^††^	16±2^††^	3.2±0.2
					(−43±5)^†††^	(−71±13)^††^	(−2±1)^†^	(0.4±2.5)
Chromanol 293B	68±4	340±14	30±2	2.4±0.2	65±9	369±11	17±2^††^	3.8±0.3^†^
	(−4±1)	(−2±5)^†^	(2±1)	(−0.3±0.1)	(−14±5)	(−21±7)	(−2±0)^†^	(−0.4±0.2)
Retigabine	53±3	310±17	36±2^††^	1.5±0.1^†††^	64±6	363±10	22±1^†^	3.2±0.3
	(−2±3)	(−5±4)^†^	(4±1)^†^	(−0.2±0.1)	(−8±2)	(−23±10)^†^	(2±0)	(−0.3±0.3)
Retigabine after reserpine^a^					63±4^†^	354±6^†^	28±2	2.3±0.2^†^
					(−9±1)	(−23±4)	(1±1)	(−0.4±0.1)
ICA-27243					61±5^†^	377±5	19±1^†††^	3.3±0.2
					(−3±5)	(−31±8)	(0±0)	(−0.1±0.2)
ICA-27243 after reserpine^a^					75±5	349±8^†^	26±2	2.9±0.2
					(−1±3)	(−25±6)	(0±1)	(−0.1±0.1)

a Reserpine was administered prior to the experiment, and the effect of reserpine combined with PBS, XE-991, retigabine or IC-27243 are therefore indicated by the differences in baselines prior to 3,4-DAP, whereas the response to PBS, XE-991 or retigabine in reserpinized SHR are shown in parenthesis below. Significant differences between the WKY and SHR control groups (^*^), between corresponding control and experimental groups (^†^) were detected as indicated.

*,† P ≤ 0.05,

**,†† P ≤ 0.01, and

††† *P ≤ 0.001*.

Effect of pre-treatment and the cardiovascular baselines after pre-treatment are shown in Table 1. In short, the ganglion blocker hexamethonium reduced MBP, HR and TPR, and MBP remained low after reserpine (tested in SHR only). However, the M2mAchR antagonist methoctramine increased baseline HR and TPR in SHR and HR in WKY (*P* ≤ 0.008).

**Figure 2:**
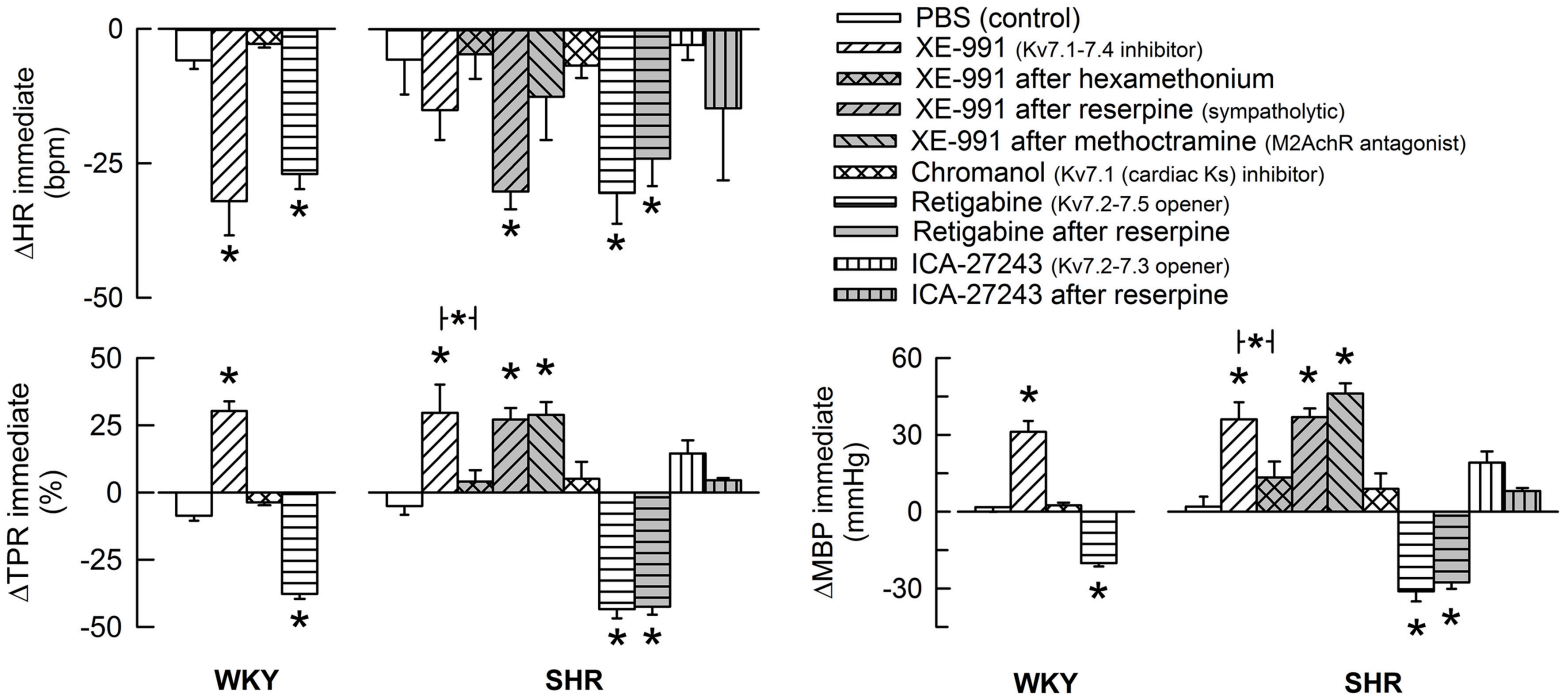
**The acute HR-, TPR-, and MBP-response to K_V_7-inhibitors and -openers**. Preferred drug selectivity is indicated by symbol legends. The drugs were given alone or, in SHR, in combination as indicated. The immediate HR- and MBP-response was recorded at the initial TPR-peak/nadir-response. Significant changes in response to channel inhibitors/openers compared to that following the sham-injection of PBS in the control groups were detected as indicated (asterix below/above columns). Involvement of ganglion transmission, sympathetic nerve norepinephrine release and M2AchR activity in the response to Kv-drug was further analyzed in SHR by prior administration of hexamethonium, reserpine or methoctramine, respectively (* in bar). * above columns—*P* ≤ 0.0167 in WKY and 0.0056 in SHR. * in brackets—*P* ≤ 0.0167 for XE-991 (none detected) and 0.025 for retigabine.

The Kv7.1-7.4-inhibitor XE-991 induced an acute (Figure 2) but transient reduction in baseline HR in WKY (*P* = 0.005). In SHR, XE-991 induced bradycardia only after reserpine (*P* < 0.001). The Kv7.1-inhibitor chromanol had no effect on resting HR in either strain (*P* = NS) (Figure 2). The Kv7.2-7.5-opener retigabine induced a transient bradycardia in both strains (*P* < 0.001), and also in reserpinized SHR (Figure 2). The Kv7.2-7.3-opener ICA-27243, alone or after reserpine, had no significant effect on baseline HR.

XE-991 induced an immediate, but transient rise in TPR (*P* ≤ 0.002) of equal magnitude in the two strains (Figure 2). The TPR-response was not different after reserpine or methoctramine but was eliminated by hexamethonium (tested in SHR). Chromanol had no significant effect on baseline TPR in either strain. The Kv7-opener retigabine precipitated a transient reduction in baseline TPR (*P* < 0.001, *P* = NS for a strain-related difference). This reduction was not influenced by reserpine (tested in SHR). The TPR-response to ICA-27243 was not statistically significant (tested in SHR). These changes in TPR were paralleled by similar changes in MBP (Figure 2). Significant changes in CO were not observed (not shown).

XE-991 induced myokymia in all rats, starting within the first minute, first as a shivering in the facial muscles, later extending into the thorax and abdomen. XE-991-induced myokymia was observed also after hexamethonium, reserpine or methoctramine. XE-991 did not evoke salivation.

### Effect of Kv7-inhibitors/openers on 3,4-DAP-induced changes in the plasma catecholamine concentrations (Table 2)

The concentration of norepinephrine in plasma collected at the end of the 3.4-DAP-observation-period was greater than that previously observed in time controls given PBS instead of 3,4-DAP in SHR (*P* ≤ 0.004) but not in WKY. The secretion of epinephrine was stimulated by the experiment itself (Berg et al., 2012), and the concentration of epinephrine in the 3,4-DAP controls did not differ from that previously observed in time-controls. The norepinephrine but not epinephrine concentration was higher in SHR than in WKY (*P* ≤ 0.003).

**Table 2:** **The concentration of catecholamines in plasma after 3,4-DAP**.

**Groups**	**WKY**		**SHR**	
	**Norepinephrine (nM)**	**Epinehrine (nM)**	**Norepinephrine (nM)**	**Epinephrine (nM)**
Time controls (PBS+PBS)^a^	0.5±0.0	7.9±1.6	1.2±0.2^†^	10.8±2.7
PBS+3,4-DAP	1.1±0.2	8.5±1.4	5.5±0.6^*^	18.5±5.2
Hexamethonium+3,4-DAP^b^			2.5±0.3^†^	3.4±1.0^†^
Reserpine+PBS+3,4-DAP			1.1±0.6^†^	11.1±2.6
Methoctramine+PBS+3,4-DAP	1.1±0.1	7.7±3.2	13.1±2.8^†^	26.9±11.8
XE-991+3,4-DAP	3.3±0.7^†^	11.6±4.2	35.6±9.1^†^	125.5±27.4^†^
Hexamethonium+XE-991+3,4-DAP			11.5±3.6^‡^^⊢^	35.9±12.6^‡^^⊢^
Reserpine+XE-991+3,4-DAP			4.4±0.9^‡^^⊢^	17.2±4.0^‡^
Methoctramine+XE-991+3,4-DAP			35.7±7.3^†^^⊢^	88.1±28.7^†^
Chromanol+3,4-DAP			6.8±2.1	35.0±23.5
Retigabine+3,4-DAP	0.8±0.2	3.9±1.4	4.7±0.5	12.3±2.2
Reserpine+retigabine+3,4-DAP			1.0±0.3^†‡^	9.7±2.1^‡^
ICA-27243+3,4-DAP			6.5±2.3	18.5±11.5
Reserpine+ICA-27243+3,4-DAP			1.4±1.0^†^	5.8±2.0

Arterial blood was collected at the end of the experiment. Significant differences between the WKY and SHR 3,4-DAP controls (*), between the 3,4-DAP-control and experimental groups within each strain (†), and in SHR between corresponding groups pre-treated with channel drug and reserpine/ hexamethonium/methoctramine+channel drug (‡), and between corresponding groups pre-treated reserpine/hexamethonium/methoctramine and reserpine/ hexamethonium/methoctramine+channel drug (^⊢^) were detected as indicated.

a *most rats in this groups was from Berg and Jensen (2011)*.

b from Berg (2015).

*,†,‡,⊢ *P < 0.05*.

Hexamethonium and reserpine alone reduced the 3,4-DAP-induced norepinephrine overflow in SHR (*P* = 0.046), and hexamethonium, but not reserpine, lowered also the concentration of epinephrine (*P* = 0.01). Methoctramine alone increased norepinephrine overflow (*P* = 0.047 compared to the controls) but not epinephrine in SHR, and had no effect in WKY. XE-991 caused a minor increase in the concentration of norepinephrine (*P* = 0.01) but not epinephrine in WKY, but greatly increased the concentration of both catecholamines in SHR (*P* ≤ 0.005). Hexamethonium and reserpine eliminated the XE-991-dependant, increased norepinephrine overflow in SHR (*P* ≤ 0.015 compared to XE-991 alone, *P* = NS compared to the controls), but the concentration remained slightly higher than that after hexamethonium or reserpine alone (*P* ≤ 0.025). Hexamethonium and reserpine also eliminated the XE-991-induced, increased secretion of epinephrine in SHR (*P* = 0.002 compared to XE-991 alone, *P* = NS compared to the controls). Methoctramine did not alter the augmenting effect of XE-991 on catecholamine release. Chromanol, retigabine and ICA-47242 did not influence the 3,4-DAP-induced changes in the plasma catecholamine concentrations.

### Effect of Kv7-inhibitors/openers on the HR-response to 3,4-DAP

**Figure 3:**
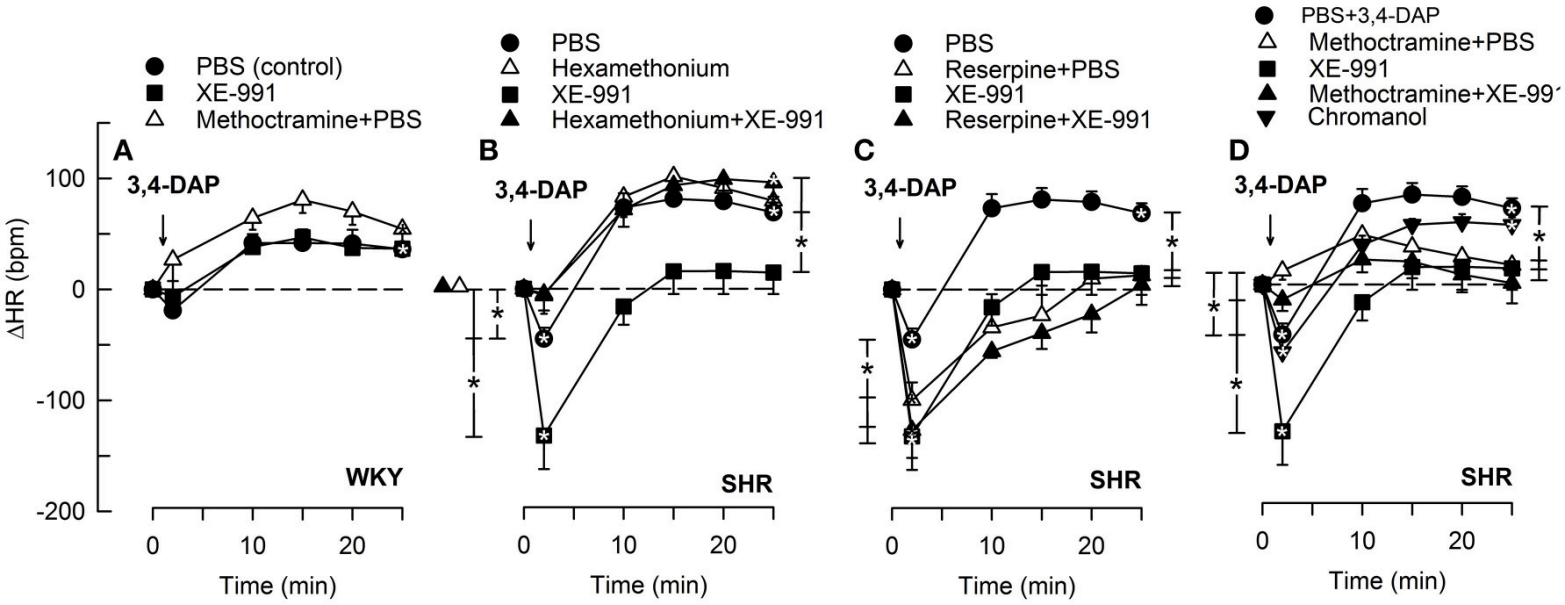
**The HR-response to 3,4-DAP after pre-treatment with the K_V_7.1-7.4-inhibitor XE-991 in WKY (A)** and SHR **(B–D)**, pre-treated as indicated by symbol legends. Significant responses (* within symbol) and group differences (* in brackets) at about 1 (recorded at the TPR-peak-response) and at 25 min were located as indicated. Comparisons were made between corresponding control and experimental groups, and between the SHR groups pre-treated with hexamethonium/reserpine/methoctramine alone and hexamethonium/reserpine/methoctramine+XE-991. *P < 0.025 after curve analyses.

As previously described (Berg, 2015), the Kv-blocker 3,4-DAP induced depolarization and in that manner activated the autonomic nervous system. 3,4-DAP therefore induced an initial bradycardia, but in SHR only (*P* < 0.001) (Figure 3). Thereafter, HR increased, and a sustained tachycardia was observed in both strains (*P* < 0.0001). ΔHR after 25 min was higher in SHR than in WKY (*P* = 0.008). Hexamethonium and methoctramine abolished the initial 3,4-DAP-induced bradycardia in SHR (Figures 3B,D), whereas reserpine eliminated the subsequent tachycardia (Figure 3C). In agreement with previous conclusions (Berg, 2015), these results demonstrated that the 3,4-DAP-induced bradycardia in SHR involved activation of cardiac, parasympathetic ganglia, whereas the tachycardia involved sympathetic nerve norepinephrine release. Methoctramine had no significant effect on the HR-response to 3,4-DAP in WKY (Figure 3A).

The M-current blocker XE-991 had no effect on the HR-response to 3,4-DAP in WKY (Figure 3A). In SHR, XE-991 enhanced the initial bradycardia and eliminated the subsequent tachycardia (*P* ≤ 0.025). The changes induced by XE-991 in SHR were further analyzed to identify possible ganglionic, parasympathetic and sympathetic involvement. The ganglion blocker hexamethonium abolished the initial bradycardia in the XE-991-treated SHR (*P* ≤ 0.003 compared to controls or XE-991 alone, Figure 3B). Hexamethonium also restored the failing tachycardia in the XE-991-treated SHR (*P* = 0.001). There was no difference between SHR groups pre-treated with XE-991 and reserpine+XE-991 (Figure 3C). Methoctramine abolished the initial 3,4-DAP-activated bradycardia in the XE-991-treated SHR (*P* = 0.008 compared to the XE-991-only group, Figure 3D), but a late tachycardia was not observed in these rats (*P* = 0.005 compared to SHR controls). Chromanol had no effect on the HR-response to 3,4-DAP in SHR (Figure 3D).

**Figure 4:**
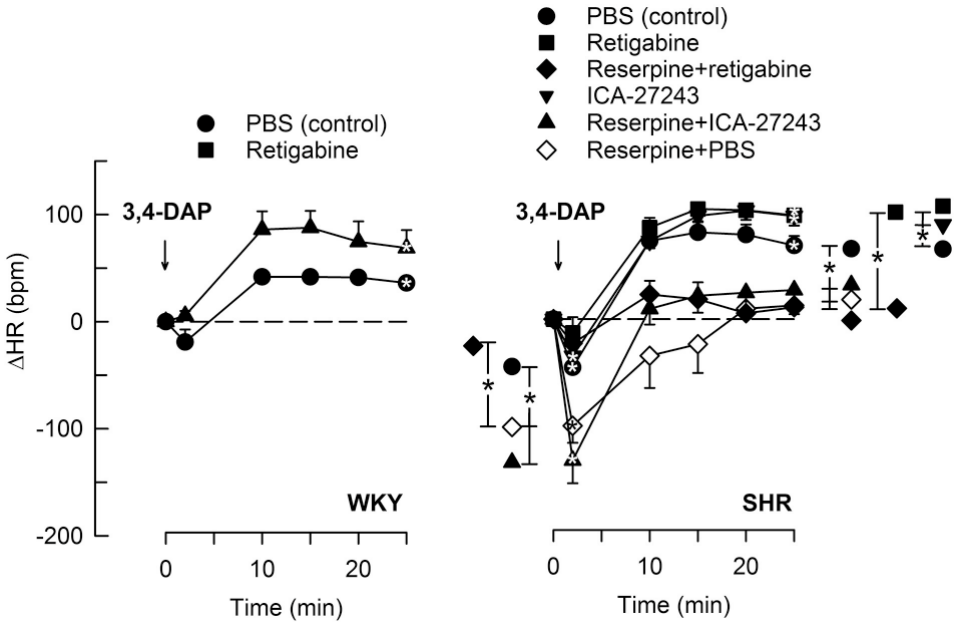
**The HR-response to 3,4-DAP after pre-treatment with Kv7-openers**. Retigabine (K_V_7.2-7.5) and ICA-27243 (K_V_7.2-7.3) were administered alone or after reserpine, as indicated by symbol legends. Significant responses (* within symbol) and group differences (* in brackets) at about 1 (recorded at the TPR-peak-response) and at 25 min were located as indicated. Comparisons were made between corresponding control and experimental groups, and between groups pre-treated with retigabine/ICA-27243 alone and reserpine+retigabine/ICA-27243. *P < 0.025 after curve analyses.

The Kv7.2-7.5-opener retigabine had no significant effect on the HR-response to 3,4-DAP in WKY (Figure 4). The initial 3,4-DAP-induced bradycardia was no longer significant in SHR after retigabine, and both retigabine and the Kv7.2-7.3-preferring ICA-27243 eliminated the enhanced bradycardia in reserpinized SHR (*P* ≤ 0.006). Both drugs also slightly enhanced the late 3,4-DAP-induced tachycardia in SHR (*P* = 0.02), but did not interfere with the reserpine-dependant inhibition on the late tachycardia.

### Effect of Kv7-inhibitors/openers on the TPR-response to 3,4-DAP

**Figure 5:**
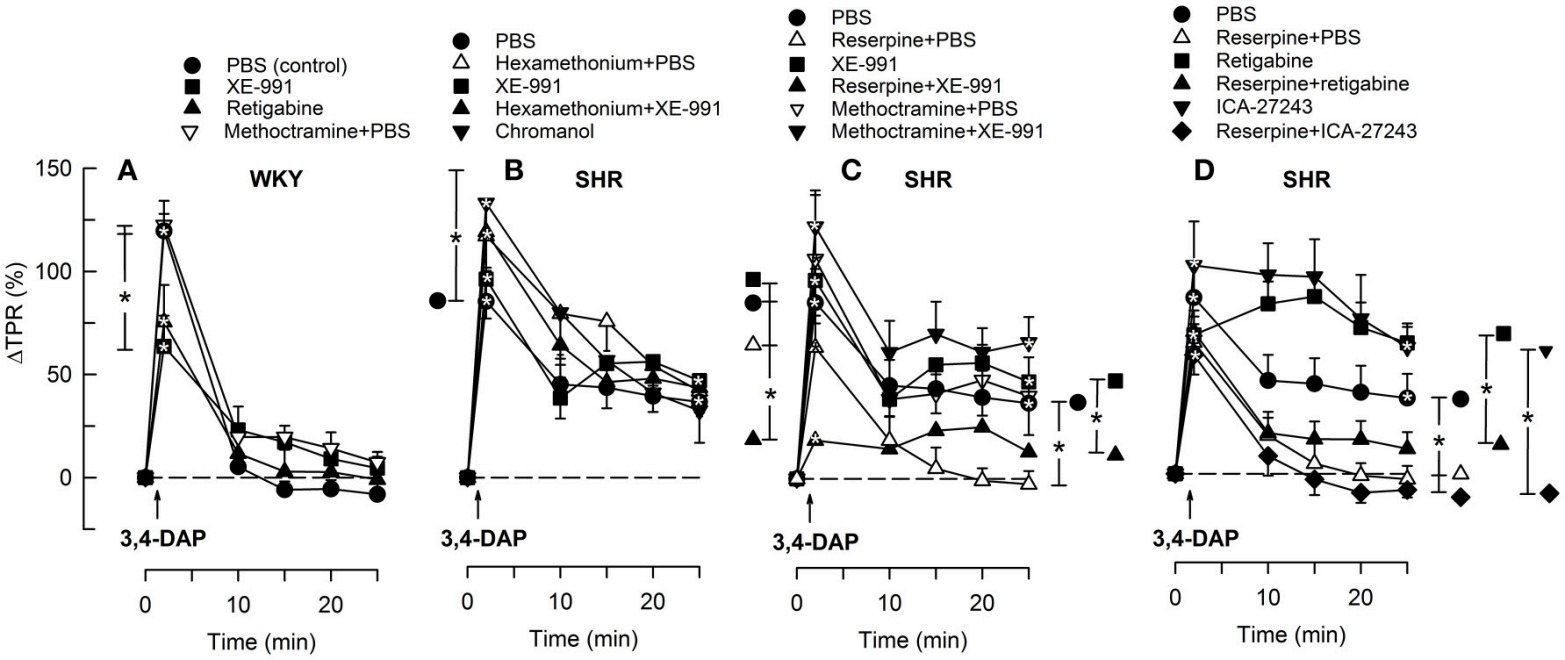
**The TPR-response to 3,4-DAP in WKY (A)** and SHR **(B–D)** after pre-treatment with Kv7-opener or -inhibitor, alone or combined with hexamethonium, reserpine or methoctramine, as indicated by symbol legends. Significant responses (* within symbol) and group differences (* in brackets) at about 1 (at the TPR-peak-response) and at 25 min were located as indicated. Comparisons were made between corresponding control and experimental groups, and between groups pre-treated with corresponding channel opener/inhibitor alone and hexamethonium/reserpine/methoctramine+channel opener/inhibitor. *P < 0.025 after curve analyses.

3,4-DAP also induced an immediate rise in TPR (*P* < 0.0001), which did not differ in the two strains (*P* = NS) (Figure 5). TPR returned quickly to baseline in WKY, but remained elevated in SHR (*P* = 0.017) and was higher than that observed in WKY (*P* = 0.007). Reserpine had no significant effect on the immediate TPR-peak-response in SHR but eliminated the elevated, late TPR-response to 3,4-DAP (*P* = 0.015, Figure 5C).

XE-991 reduced the TPR-peak-response to 3,4-DAP in WKY (*P* = 0.015, Figure 5A) but had no significant effect in SHR (Figure 5B). XE-991 did not alter the late TPR-response to 3,4-DAP in either strain (Figures 5A,B). Kv7 inhibitor/openers had otherwise no effect on the TPR-response to 3,4-DAP, and did not interfere with the inhibitory effect of reserpine in SHR (Figures 5C,D). However, reserpine+XE-991 clearly reduced the TPR-response throughout the 3,4-DAP-observation-period in SHR (Figure 5C) The changes in MBP largely followed these changes in TPR (not shown).

### Effect of Kv7-inhibitors/openers on 3,4-DAP-induced salivation (Table 3)

3,4-DAP activated secretion of saliva with a greater effect in WKY than in SHR (*P* ≤ 0.045). Salivation in SHR was reduced by 81 and 74% after reserpine and hexamethonium, respectively (*P* ≤ 0.014). Methoctramine enhanced the 3,4-DAP-induced salivation in WKY (*P* = 0.017), but had no effect in SHR. XE-991 and chromanol had no significant effect on saliva volume. The channel opener retigabine reduced the 3,4-DAP-induced salivation in WKY (*P* = 0.009). Retigabine and ICA-27243 had no significant effect in SHR. XE-991, retigabine and ICA-27243 did not interfere with the lowering effect of reserpine, but XE-991 eliminated the reduction induced by hexamethonium (tested in SHR only). Salivation in SHR pre-treated with methoctramine+XE-991 was not different from that in the controls or after methoctramine alone.

**Table 3:** **Effect of K_V_7-inhibitors/openers on 3,4-DAP-induced salivation**.

**Groups**	**Saliva volume (μl)**	
	**WKY**	**SHR**
PBS+3,4-DAP (control)	394±96	131±52^*^
Hexamethonium+3,4-DAP		34±2^†^
Reserpine+PBS+3,4-DAP		25±11^†^
Methoctramine+PBS+3,4-DAP	737±70^†^	124±25
XE-991+3,4-DAP	319±118	72±27
Hexamethonium+XE-991+3,4-DAP		177±67
Reserpine+XE-991+3,4-DAP		49±22^†^
Methoctramine+XE-991+3,4-DAP		176±51
Chromanol+3,4-DAP		114±12
Retigabine+3,4-DAP	115±34^†^	71±50
Reserpine+retigabine+3,4-DAP		14±13^†^
ICA-27243+3,4-DAP		72±21
Reserpine+ICA-27243+3,4-DAP		31±9^†^

Salivation does not occur in anesthetized rats, unless stimulated, as here by the 3,4-DAP-induced activation of the autonomic nervous system. Significant differences between the WKY and SHR control groups (*), between corresponding control and experimental groups (†) and between corresponding groups pre-treated with XE-991/retigabine/ICA-27243 alone and reserpine/hexamethonium/methoctramine+XE-991/retigabine/ICA-27243 (none detected) were detected as indicated.

*,† *P < 0.05*.

## Discussion

The main result in the present study was that inhibition of hyperpolarizing M-channels, most likely Kv7.2-7.3, enhanced parasympathetic ganglion transmission and vagal control of HR in SHR, and also increased sympathetic nerve and adrenal catecholamine release in this strain, but had little effect in WKY. Vascular M-channels, i.e., Kv7.4 and possibly also 7.5, had a vasodilatory effect on resting TPR in both strains.

### Role of M-currents in catecholamine release

In unstimulated rats, the plasma norepinephrine concentrations are low due to re-uptake through the norepinephrine re-uptake transporter, and differences in release are difficult to demonstrate (Berg et al., 2012; Berg and Jensen, 2013). Autonomic nerve transmitter release was therefore stimulated by depolarization, induced by inhibition of presynaptic Kv by 3,4-DAP. 3,4-DAP itself does not inhibit *I*_M_ (Goh et al., 1989). Previous analyses showed that 3,4-DAP stimulated release primarily in the parasympathetic ganglia and in sympathetic nerve terminals (Berg, 2015; Figure 1). Inhibition of the stabilizing M-channels with XE-991, but not chromanol, induced a large increase in norepinephrine overflow to plasma and epinephrine secretion in SHR, but had little effect in WKY. M-currents, most likely Kv7.2-7.3, therefore strongly opposed the release of both norepinephrine and epinephrine in SHR, but not in WKY. As expected, the XE-991-dependant stimulation of norepinephrine release was eliminated by reserpine. Unexpectedly, reserpine also abolished the increased secretion of epinephrine, possibly indicating that sympathetic nerves or the high level of circulating norepinephrine stimulated the secretion of epinephrine. However, it could not be excluded that this effect of reserpine involved a central action. Also hexamethonium eliminated the XE-991-dependant increased catecholamine secretion. The effect on epinephrine secretion was explained by inhibition of the adrenal, postsynaptic nAchR, a prime activator of adrenal epinephrine secretion. This conclusion was confirmed by that hexamethonium also alone reduced the secretion of epinephrine. The effect of hexamethonium on the increased norepinephrine overflow after XE-991+3,4-DAP as well as after 3,4-DAP alone, may result from inhibition of presynaptic nAchR, which facilitate the release of norepinephrine (Haass and Kübler, 1997). This observation suggested that *I*_M_ depended on Ach, which may be released from the preganglionic neuron or from adjacent parasympathetic nerve fibers (Figure 1). The two-fold increase in the plasma norepinephrine but not epinephrine concentration in SHR pre-treated with methoctramine alone was compatible with inhibition of presynaptic M2mAchR, known to inhibit norepinephrine release from sympathetic nerves (Jumblatt and Hackmiller, 1994). A similar effect was not observed in WKY. Presynaptic M2mAchR evidently did not influence sympathetic nerve M-currents since methoctramine did not hamper the increased catecholamine concentrations in XE-991-treated SHR.

### Role of M-currents in resting HR-control

Since hyperpolarizing M-currents oppose nerve excitability, the fall in resting HR in response to the K_V_7.1-7.4-preferring inhibitor XE-991 (Yeung et al., 2007) in WKY was likely to reflect an increased parasympathetic activity, rather than a decrease in the sympathetic control of HR. A role of cardiac K_V_7.1 (K_S_), which contributes to repolarization in the heart, was excluded, since chromanol did not influence resting HR. It was therefore concluded that members of the Kv7-family, most likely K_V_7.2-7.3, were open in WKY and sustained resting HR by inhibiting vagal transmission.

XE-991 destabilized also sympathetic nerves, and an XE-991-induced, parasympathetic bradycardia was detected in SHR only after the sympathetic nerve terminals were depleted of norepinephrine by reserpine. Thus, the stimulating effect of XE-991 on resting, parasympathetic HR-control was hampered in SHR by the simultaneous increase in sympathetic nerve activity. This conclusion was supported by that XE-991 increased 3,4-DAP-induced catecholamine release predominantly in SHR, as discussed above. This *I*_M_-dependant autonomic imbalance in SHR may explain the high resting HR in this strain.

The Kv7-opener retigabine (K_V_7.2-7.5) reduced resting HR equally in the two strains, compatible with a reduced neuronal and/or adrenal catecholamine release. However, retigabine induced bradycardia also in reserpinized SHR, which may be due to that adrenergic HR-control after reserpine relied on epinephrine. However, retigabine may stimulate GABA synthesis and GABA-activated Cl^−^ channels (Kapetanovic et al., 1995; Rundfeldt and Netzer, 2000), which may in the central nervous system influence resting HR. The latter explanation was supported by the fact that ICA-27243 (K_V_7.2-7.3-preferring) failed to lower resting HR in SHR.

### Role of M-currents in HR-control in the stimulated heart

M-channels are likely to be more open during nerve depolarization. Through changes in HR, 3,4-DAP-induced neuronal depolarization offered the opportunity to study the role of M-channels in not only sympathetic but also parasympathetic excitation. XE-991 enhanced the immediate 3,4-DAP-induced bradycardia. This effect was eliminated by methoctramine, indicating activation of parasympathetic, postsynaptic mAchR (Figure 1), which are of the M2-sutype in the heart (Wess et al., 1988) Furthermore, reserpine, by eliminating the counter-acting effect of norepinephrine release, enhanced the 3,4-DAP-induced bradycardia, but did not alter the effect of XE-991 on the immediate HR-response to 3,4-DAP. Also hexamethonium abolished the increased bradycardia in XE-991-treated SHR. These observations located the stimulating effect of XE-991 to the parasympathetic ganglia. It was therefore concluded that M-currents hampered vagal control of HR in SHR, apparently by hampering parasympathetic ganglion transmission. XE-991 had no effect on the HR-response to 3,4-DAP in WKY, indicating that M-currents did not hamper vagal function in normotensive rats.

An inhibitory effect of *I*_M_ on parasympathetic signaling, was further supported by the absence of an immediate, 3,4-DAP-induced bradycardia in SHR pre-treated with the Kv7-opener retigabine, and, even more, by that retigabine and ICA-27243 eliminated the enhanced bradycardia in reserpinized SHR. The latter result excluded that the role of M-currents in the initial 3,4-DAP-induced bradycardia involved reductions in sympathetic activity. In agreement with a reduced parasympathetic activation in the presence of M-channel openers, both Kv-openers also enhanced the late 3,4-DAP-induced tachycardia in SHR. Like XE-991, the effect of retigabine on the HR-response to 3,4-DAP in WKY was not statistically significant, compatible with a role of M-currents in parasympathetic and sympathetic nerve excitability in SHR only.

Methoctramine and XE-991, separately or combined, also eliminated the late 3,4-DAP-induced tachycardia in SHR, in spite of high levels of circulating catecholamines in SHR given XE-91. The reasons underlying these observations were not clear, and similar effects were not observed after methoctramine or XE-991 in WKY. However, the inhibitory effect of XE-991 may be due to a dominating effect of the XE-991-induced parasympathetic activation. This conclusion was supported by that the tachycardia was restored by hexamethonium, which through ganglion blockade will eliminate the XE-991-activated parasympathetic excitation.

Neuronal M-currents are primarily mediated through K_V_7.2-7.3 (Wang et al., 1998), compatible with the present Kv7-inhibitor profiles, although the presents results did not exclude a role of Kv7.4. Neuronal M-channels are clustered in the axonal initial segment and in the nodes of Ranvier (Pan et al., 2006; Figure 1). However, M-channels have also been detected in the preganglionic vagal nerve fibers together with the 4-aminopyridine-sensitive Kv1.1 (Glasscock et al., 2012). The inhibitory effect of the nAChR antagonist hexamethonium on the XE-991-stimulated parasympathetic bradycardia may be interpreted in support of both a preganglionic and a postganglionic M-channel localization in the parasympathetic branch (Figure 1). However, although hexamethonium blocked the effect of XE-991 on norepinephrine release, it did not prevent the late, 3,4-DAP-induced, norepinephrine-dependant tachycardia in SHR. The M-channels may therefore possibly have a postganglionic localization in the sympathetic branch.

### Role of M-currents in TPR-control

Vasodilatory K_V_7.1, 7.4, and 7.5 have been demonstrated in rat arterial VSMC (Mackie et al., 2008), although an additional presence in the endothelium could not be excluded. XE-991 increased, and retigabine reduced resting TPR, whereas the Kv7.1- and 7.2-7.3-preferring drugs chromanol and ICA-27243 had no effect. These responses were not different in reserpinized SHR. It was therefore concluded that M-channels, most likely K_V_7.4 and possibly also 7.5, controlled VSMC tension, independent of norepinephrine release. No strain-related difference was detected. This result differed from that previously observed in *in vitro* studies, where Kv7.4-mediated arterial vasodilatation was found to be reduced in SHR (Jepps et al., 2011; Chadha et al., 2012). The vasoconstrictory TPR-response to XE-991 was also not influenced by M2AchR antagonist, indicating that the VSMC M-currents were not activated by M2AchR. Methoctramine itself increased resting TPR in SHR, possibly due to increased norepinephrine release, compatible with that observed after 3,4-DAP-stimulation, as discussed above. Increased norepinephrine release may also explain why methoctramine increased resting HR in both strains, but this increase may also be explained by inhibition of postsynaptic M2mAchR, which mediate vagal function in the heart. The XE-991-induced vasoconstriction observed in SHR was totally eliminated by hexamethonium. This observation may suggest an inhibitory effect of nAchR on M-currents in VSMC, in accordance with the transient rise in TPR following injection of nicotine (Berg, 2015). However, other mechanisms may be involved.

The injection of 3,4-DAP precipitated an immediate rise in TPR in both strains. This was likely to primarily result from inhibition of 3,4-DAP-sensitive Kv in the VSMC (Berg, 2002, 2003). The TPR-response was transient in WKY, but sustained in SHR, and the late vasoconstriction in SHR depended on the 3,4-DAP-induced release of norepinephrine since it was eliminated by reserpine. The Kv7-inhibitors/openers had no clear, decipherable effect on the TPR-response to 3,4-DAP, possibly because *I*_M_ were likely to influence VSMC contractility through the same mechanisms as 3,4-DAP, i.e., altering the VSMC membrane potential by altering K^+^ conductance, thus influencing Ca_V_ and the entry of Ca^2+^. 3,4-DAP was therefore not suitable for studying the impact of M-currents on norepinephrine-stimulated vasoconstriction.

### Role of M-currents in 3,4-DAP-induced salivation

The 3,4-DAP-induced salivary flow apparently depended on parasympathetic activation since it was almost totally abolished by atropine (Berg, 2015) but not inhibited by methoctramine, in accordance with that parasympathetic salivation is mediated primarily by M3, but also by M1 and M4 mAchR (Bymaster et al., 2003). However, salivation was also reduced by reserpine in SHR and by the β-adrenoceptor blocker nadolol in both strains (Berg, 2015), indicating dependence of sympathetic nerve activation. 3,4-DAP-induced saliva also contained a high concentration of kallikrein in SHR, the secretion of which depends on α-adrenoceptor activation (Berg, 2015). The reduced salivation observed after hexamethonium, may therefore also be explained by inhibition of presynaptic nAchR, which facilitate release of norepinephrine (Haass and Kübler, 1997). The augmenting effect of methoctramine on 3,4-DAP-induced salivation in WKY was likely to result from inhibition of presynaptic M2mAchR, which inhibit norepinephrine release from sympathetic nerves (Jumblatt and Hackmiller, 1994). The 3,4-DAP-induced salivation therefore appeared to depend on sympathetic nerve activation, modulated by presynaptic nAchR or mAchR, activated by the 3,4-DAP-induced release of Ach. In spite of the XE-991-induced increased catecholamine release, only retigabine reduced salivation and in WKY only, explained by reduced neuronal excitability.

### Myokymia

XE-991 activated myokymia in both strains, fully compatible with the antiepileptic effect of retigabine by opening K_V_7.2-7.3 (Wickenden et al., 2000). The impact of *I*_M_ on autonomic control in SHR revealed by the present experiments, may indicate a need for analyses of possible beneficial or unwanted side-effects of *I*_M_-openers as antiepileptic therapy in cardiovascular risk patients. It may also be considered if sudden death in epilepsy may involve Kv7-dependant cardiac autonomic dysfunctions.

## Conclusions

The present study showed for the first time that M-channels stabilized parasympathetic nerves and inhibited vagal control of HR in SHR but not WKY. M-channels also opposed release of norepinephrine and epinephrine, and, when inhibited, massive catecholamine release was observed in response to nerve stimulation, but only in SHR. The up-regulation of the stabilizing M-currents in SHR may be a primary defect causing sympathetic tone to rise, but may also be a compensation for an up-regulated central sympathetic output. The M-channel-dependent changes observed in SHR are likely to play an important role in the dysfunctional autonomic control of HR in hypertension and possibly also in heart failure, myocardial infarction and atrial fibrillation. In addition, M-channels were demonstrated to play a clear role in down-regulating VSMC resting tension and TPR, but with no strain-related difference detected. The M-channels controlling neuronal excitation were likely to be Kv7.2-7.3, whereas Kv7.4, possibly also Kv7.5, regulated vascular tension. Thus, channel-specific drugs may allow selective manipulation of neuronal and vascular M-currents, and may have a future as antihypertensive or antiarrhythmic therapeutics.

## Author contributions

The author confirms being the sole contributor of this work and approved it for publication.

## Funding

This work was supported by The Norwegian Council on Cardiovascular Diseases and Anders Jahre's Fond.

### Conflict of interest statement

The author declares that the research was conducted in the absence of any commercial or financial relationships that could be construed as a potential conflict of interest. The reviewer JLDBA and handling Editor declared their shared affiliation, and the handling Editor states that the process nevertheless met the standards of a fair and objective review.

## References

[B1] Berg T. (2002). Analysis of the pressor response to the K^+^ channel inhibitor 4-aminopyridine. Eur. J. Pharmacol. 452, 325–337. 10.1016/S0014-2999(02)02306-3 12359274

[B2] Berg T. (2003). The vascular response to the K^+^ channel inhibitor 4-aminopyridine in hypertensive rats. Eur. J. Pharmacol. 466, 301–310. 10.1016/S0014-2999(03)01555-3 12694813

[B3] Berg T. (2005). Increased counteracting effect of eNOS and nNOS on an α_1_-adrenergic rise in total peripheral vascular resistance in spontaneous hypertensive rats. Cardiovasc. Res. 67, 736–744. 10.1016/j.cardiores.2005.04.006 15907821

[B4] Berg T. (2015). Voltage-sensitive K^+^ channels inhibit parasympathetic ganglion transmission and vagal control of heart rate in hypertensive rats. Front. Neurol. 6:260. 10.3389/fneur.2015.00260 26696959 PMC4672051

[B5] Berg T. Jensen J. (2011). Simultaneous parasympathetic and sympathetic activation reveals altered autonomic control of heart rate, vascular tension, and epinephrine release in anesthetized hypertensive rats. Front. Neurol. 2:71. 10.3389/fneur.2011.00071 22131984 PMC3222849

[B6] Berg T. Jensen J. (2013). Tyramine reveals failing α_2_-adrenoceptor control of catecholamine release and total peripheral vascular resistance in hypertensive rats. Front. Neurol. 4:19. 10.3389/fneur.2013.00019 23450822 PMC3584258

[B7] Berg T. Walaas S. I. Roberg B. Å. Huynh T. T. Jensen J. (2012). Plasma norepinephrine in hypertensive rats reflects α_2_-adrenoceptor release control only when re-uptake is inhibited. Front. Neurol. 3:160. 10.3389/fneur.2012.00160 23162530 PMC3492874

[B8] Bibevski S. Dunlap M. E. (2011). Evidence for impaired vagus nerve activity in heart failure. Heart Fail. Rev. 16, 129–135. 10.1007/s10741-010-9190-6 20820912

[B9] Binkley P. F. Nunziata E. Haas G. J. Nelson S. D. Cody R. J. (1991). Parasympathetic withdrawal is an integral component of autonomic imbalance in congestive heart failure: demonstration in human subjects and verification in a paced canine model of ventricular failure. J. Am. Coll. Cardiol. 18, 464–472. 10.1016/0735-1097(91)90602-6 1856414

[B10] Brown D. A. Passmore G. M. (2009). Neural KCNQ (Kv7) channels. Br. J. Pharmacol. 156, 1185–1195. 10.1111/j.1476-5381.2009.00111.x 19298256 PMC2697739

[B11] Bymaster F. P. Carter P. A. Yamada M. Gomeza J. Wess J. Hamilton S. E.. (2003). Role of specific muscarinic receptor subtypes in cholinergic parasympathomimetic responses, *in vivo* phosphoinositide hydrolysis, and pilocarpine-induced seizure activity. Eur. J. Neurosci. 17, 1403–1410. 10.1046/j.1460-9568.2003.02588.x 12713643

[B12] Chadha P. S. Zunke F. Zhu H. L. Davis A. J. Jepps T. A. Olesen S. P.. (2012). Reduced KCNQ4-encoded voltage-dependent potassium channel activity underlies impaired β-adrenoceptor-mediated relaxation of renal arteries in hypertension. Hypertension 59, 877–884. 10.1161/HYPERTENSIONAHA.111.187427 22353613

[B13] Glasscock E. Qian J. Kole M. J. Noebels J. L. (2012). Transcompartmental reversal of single fibre hyperexcitability in juxtaparanodal Kv1.1-deficient vagus nerve axons by activation of nodal KCNQ channels. J. Physiol. 590, 3913–3926. 10.1113/jphysiol.2012.235606 22641786 PMC3476640

[B14] Goh J. W. Kelly M. E. Pennefather P. S. (1989). Electrophysiological function of the delayed rectifier (IK) in bullfrog sympathetic ganglion neurones. Pflügers Arch. 413, 482–486. 10.1007/BF00594177 2787017

[B15] Haass M. Kübler W. (1997). Nicotine and sympathetic neurotransmission. Cardiovasc. Drugs Ther. 10, 657–665. 10.1007/BF00053022 9110108

[B16] Jepps T. A. Chadha P. S. Davis A. J. Harhun M. I. Cockerill G. W. Olesen S. P.. (2011). Downregulation of Kv7.4 channel activity in primary and secondary hypertension. Circulation 124, 602–611. 10.1161/CIRCULATIONAHA.111.032136 21747056

[B17] Jumblatt J. E. Hackmiller R. C. (1994). M2-type muscarinic receptors mediate prejunctional inhibition of norepinephrine release in the human iris-ciliary body. Exp. Eye Res. 58, 175–180. 10.1006/exer.1994.1005 7512511

[B18] Kapetanovic I. M. Yonekawa W. D. Kupferberg H. J. (1995). The effects of D-23129, a new experimental anticonvulsant drug, on neurotransmitter amino acids in the rat hippocampus *in vitro*. Epilepsy Res. 22, 167–173. 10.1016/0920-1211(95)00050-X 8991783

[B19] Kleiger R. E. Miller J. P. Bigger J. T. Jr. Moss A. J. (1987). Decreased heart rate variability and its association with increased mortality after acute myocardial infarction. Am. J. Cardiol. 59, 256–262. 10.1016/0002-9149(87)90795-8 3812275

[B20] Levy D. Garrison R. J. Savage D. D. Kannel W. B. Castelli W. P. (1990). Prognostic implications of echocardiographically determined left ventricular mass in the Framingham Heart Study. N. Engl. J. Med. 322, 1561–1566. 10.1056/NEJM199005313222203 2139921

[B21] Mackie A. R. Brueggemann L. I. Henderson K. K. Shiels A. J. Cribbs L. L. Scrogin K. E.. (2008). Vascular KCNQ potassium channels as novel targets for the control of mesenteric artery constriction by vasopressin, based on studies in single cells, pressurized arteries, and *in vivo* measurements of mesenteric vascular resistance. J. Pharmacol. Exp. Ther. 325, 475–483. 10.1124/jpet.107.135764 18272810 PMC2597077

[B22] Palatini P. (1999). Elevated heart rate as a predictor of increased cardiovascular morbidity. J. Hypertens. 17(Suppl. 3), S3–S10. 10489092

[B23] Pan Z. Kao T. Horvath Z. Lemos J. Sul J. Y. Cranstoun S. D.. (2006). A common ankyrin-G-based mechanism retains KCNQ and NaV channels at electrically active domains of the axon. J. Neurosci. 26, 2599–2613. 10.1523/JNEUROSCI.4314-05.2006 16525039 PMC6675151

[B24] Park H. W. Shen M. J. Lin S. F. Fishbein M. C. Chen L. S. Chen P. S. (2012). Neural mechanisms of atrial fibrillation. Curr. Opin. Cardiol. 27, 24–28. 10.1097/HCO.0b013e32834dc4e8 22139702 PMC3279730

[B25] Qi J. Zhang F. Mi Y. Fu Y. Xu W. Zhang D.. (2011). Design, synthesis and biological activity of pyrazolo[1,5-a]pyrimidin-7(4H)-ones as novel Kv7/KCNQ potassium channel activators. Eur. J. Med. Chem. 46, 934–943. 10.1016/j.ejmech.2011.01.010 21296466

[B26] Rundfeldt C. Netzer R. (2000). Investigations into the mechanism of action of the new anticonvulsant retigabine. Interaction with GABAergic and glutamatergic neurotransmission and with voltage gated ion channels. Arzneimittelforschung 50, 1063–1070. 10.1055/s-0031-1300346 11190770

[B27] Schlaich M. P. Kaye D. M. Lambert E. Sommerville M. Socratous F. Esler M. D. (2003). Relation between cardiac sympathetic activity and hypertensive left ventricular hypertrophy. Circulation 108, 560–565. 10.1161/01.CIR.0000081775.72651.B6 12847071

[B28] Thrasher T. N. (2005). Baroreceptors, baroreceptor unloading, and the long-term control of blood pressure. Am. J. Physiol. Regul. Integr. Comp. Physiol. 288, R819–R827. 10.1152/ajpregu.00813.2004 15793035

[B29] Vlcková E. Stolc S. (1990). 3,4-Diaminopyridine induced stimulus-bound repetitive firing in frog sympathetic ganglion: no changes in postsynaptic membrane excitability. Physiol. Bohemoslov. 39, 519–531. 1966500

[B30] Wang H. S. Pan Z. Shi W. Brown B. S. Wymore R. S. Cohen I. S.. (1998). KCNQ2 and KCNQ3 potassium channel subunits: molecular correlates of the M-channel. Science 282, 1890–1893. 10.1126/science.282.5395.1890 9836639

[B31] Wess J. Angeli P. Melchiorre C. Moser U. Mutschler E. Lambrecht G. (1988). Methoctramine selectively blocks cardiac muscarinic M2 receptors *in vivo*. Naunyn Schmiedebergs. Arch. Pharmacol. 338, 246–249. 10.1007/bf00173395 3057387

[B32] Wickenden A. D. Yu W. Zou A. Jegla T. Wagoner P. K. (2000). Retigabine, a novel anti-convulsant, enhances activation of KCNQ2/Q3 potassium channels. Mol. Pharmacol. 58, 591–600. 10.1124/mol.58.3.591 10953053

[B33] Yang Z. Shi G. Li C. Wang H. Liu K. Liu Y. (2004). Electrophysiologic effects of nicorandil on the guinea pig long QT1 syndrome model. J. Cardiovasc. Electrophysiol. 15, 815–820. 10.1046/j.1540-8167.2004.03632.x 15250869

[B34] Yeung S. Y. Pucovský V. Moffatt J. D. Saldanha L. Schwake M. Ohya S.. (2007). Molecular expression and pharmacological identification of a role for K(v)7 channels in murine vascular reactivity. Br. J. Pharmacol. 151, 758–770. 10.1038/sj.bjp.0707284 17519950 PMC2014117

